# Emergence of spontaneous anticipatory hand movements in a probabilistic environment

**DOI:** 10.2478/v10053-008-0132-y

**Published:** 2013-06-17

**Authors:** Pernille Bruhn

**Affiliations:** Department of Psychology, Aarhus University, Denmark

**Keywords:** anticipation, prediction, probabilistic spatial cuing, statistical learning, computer mouse tracking

## Abstract

In this article, we present a novel experimental approach to the study of anticipation in probabilistic cuing. We implemented a modified spatial cuing task in which participants made an anticipatory hand movement toward one of two probabilistic targets while the (*x*, *y*)-computer mouse coordinates of their hand movements were sampled. This approach allowed us to tap into anticipatory processes as they occurred, rather than just measuring their behavioral outcome through reaction time to the target. In different conditions, we varied the participants’ degree of certainty of the upcoming target position with probabilistic pre-cues. We found that participants initiated spontaneous anticipatory hand movements in all conditions, even when they had no information on the position of the upcoming target. However, participants’ hand position immediately before the target was affected by the degree of certainty concerning the target’s position. This modulation of anticipatory hand movements emerged rapidly in most participants as they encountered a constant probabilistic relation between a cue and an upcoming target position over the course of the experiment. Finally, we found individual differences in the way anticipatory behavior was modulated with an uncertain/neutral cue. Implications of these findings for probabilistic spatial cuing are discussed.

## Introduction

The anticipation of upcoming events is proposed to be a fundamental and pervasive mechanism of the brain, and anticipation is thought to be crucial in a large array of cognitive functions from perception through action (e.g., Anokhin, 1974; Bubic, von Cramon, & Schubotz, 2010; Llinás, 2001; Pezzulo, Hoffmann, & Falcone, 2007; Raichle & Gusnard, 2005; Summerfield & Egner, 2009). Indeed, there is ample evidence for the existence of anticipatory activity in the brain: Neural activity similar to the activity involved in actually perceiving a given event can be observed before the given event when participants are expecting this event to happen in the immediate future (Bastiaansen & Brunia, 2001; Carlsson, Petrovic, Skare, Petersson, & Ingvar, 2000; Chawla, Rees, & Friston, 1999; Langner et al., 2011; Luks & Simpson, 2004; Macaluso, Eimer, Frith, & Driver, 2003; Shulman, d’Avossa, Tansy, & Corbetta, 2002; Sylvester, Shulman, Jack, & Corbetta, 2007; Voisin, Bidet-Caulet, Bertrand, & Fonlupt, 2006). Moreover, a lot of studies have consistently found lower reaction time to expected compared to unexpected events, indicating that anticipation has significant behavioral effects (Bruhn & Bundesen, 2012; Dykes & Pascal, 1981; Eriksen & Yeh, 1985; Feigenberg, 2008; Kingstone, 1992; Kingstone & Klein, 1991; Krueger, 1970; Mattes, Ulrich, & Miller, 2002; Miller & Anbar, 1981; Posner, 1980).

On a very basic level, the mechanism of anticipation can be described as the capacity of the central nervous system for “modelling” the course and outcome of future events on the basis of past experience with recurring phenomena (Anokhin, 1974; Sokolov, Spinks, Näätänen, & Lyytinen, 2002). Anokhin (1974) argues that it is a basic adaptive mechanism that when an organism repeatedly encounters a specific sequence of events (e.g., of the type “*A* → *B*”) then event *A* will eventually acquire *signal value* for the future event *B*, and the organism will start to anticipate *B* when it encounters *A* (i.e., before *B* actually occurs). However, the relationship between a given *signaling* event (*A*) and the *signaled* upcoming event (*B*) is not always a perfect causal relationship, that is, the predictive relation between many natural events is *probabilistic* rather than deterministic (e.g., Feigenberg, 1969). Thus, for anticipation to be functional in a non-deterministic world, the probability of a future event should be taken into account and shape anticipatory processes in a systematic way.

The present study investigated how repeated experience with probabilistic sequences of events shapes anticipatory processes. In our experimental task, participants moved the computer mouse from an initial position at the bottom of the computer screen to click on a target that would occur either on the left or on the right upper part of the screen. (This basic setup was inspired by previous studies which introduced the use of mouse tracking in cognitive tasks; see McKinstry, Dale, & Spivey, 2008; Spivey, Grosjean, & Knoblich, 2005.) Before the presentation of the target, one of five possible cues was presented centrally on the screen. Unbeknownst to the participants, each of these cues represented a given conditional probability of the target subsequently occurring on the left or on the right side of the screen. Hence, each cue contained information on how certain the participants could be about the position of the upcoming target on the current trial. This degree of certainty concerning the upcoming target’s position was varied over three mixed conditions: (a) a *certain* condition (100%-valid cue), (b) a *semi-certain* condition (75%-valid cue), and (c) an *uncertain* condition (neutral/uninformative cue). The specific probabilistic relation between a given cue and a given subsequent target position was not given explicitly to the participants. However, we expected that the participants would gradually “pick up” these probabilistic regularities and that this would become reflected in their anticipatory hand movements toward the targets. Studies on statistical learning have shown that even infants are able to learn the implicit probabilistic structure of sequences (of, e.g., different visual forms) and the spatiotemporal occurrence of such forms (Kirkham, Slemmer, & Johnson, 2002; Kirkham, Slemmer, Richardson, & Johnson, 2007). In our study, we were not concerned with whether participants’ (potential) “learning” of the probabilistic predictive relationship between cues and targets was implicit or explicit (see Dale, Duran, & Morehead, 2012, for a discussion of implicit and explicit learning related to anticipatory behavior in a statistical learning task). Simply, we wanted to explore if, how quickly, and in which way emerging probability-based degrees of certainty modulate anticipatory behavior.

Because anticipation by definition takes place before the event it is directed at, traditional outcome-based measures such as reaction time and accuracy only offer an indirect, post hoc measure of anticipation. By measuring participants’ hand movements before the target occurred we were able to tap into anticipatory processes as they occurred, rather than just indirectly measuring anticipation through target responses.

## Methods

### Participants

Eleven undergraduate students (seven females and four males between 20 and 22 years old, *M*_age_ = 21.2, **SD** = 0.7) from the Department of Psychology, Aarhus University participated as unpaid volunteers. Data from two of the 11 participants were excluded from the analysis as these participants reported that they had voluntarily ignored the cues throughout the experiment.

### Apparatus

The experiment was programmed and run using *E-Prime* (Psychology Software Tools, Inc., Pittsburgh, PA). Stimuli were presented on a 15” computer monitor (800 × 600 pixels), and responses were given by moving a standard computer mouse and clicking its left button. The mouse cursor settings used were Windows’ standard settings.

### Experimental set-up and stimuli

**Figure 1.**
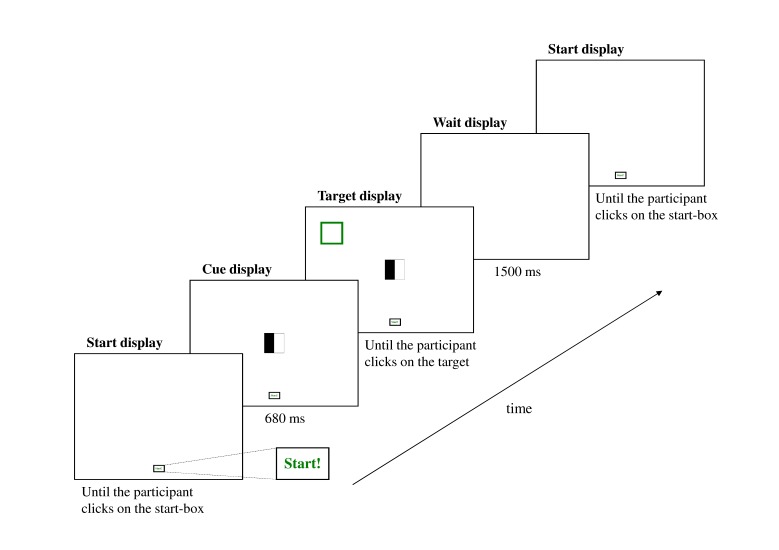
The sequence and positioning of stimuli in a single trial. The start box was a 50 × 30 pixels rectangle with the word “Start!” written in green inside. The center of the rectangle was positioned at the horizontal midline, 35 pixels above the bottom of the screen. The cues were 100 × 100 pixels black and white squares positioned with their center at the intersection of the horizontal and the vertical midlines of the screen. The target was a 100 × 100 pixels square with green borders positioned with its center 170 pixels from the upper left or right corner of the screen for the left- and right-side targets, respectively. The relative sizes and positions of the objects in this figure roughly correspond to those of the experiment. The text below each screen diagram indicates the duration of the screen display.

An example of a single trial is shown in Figure 1. At the beginning of each trial, a start box was displayed at the bottom of the screen. The mouse cursor was visible and locked to a position in the middle of the start box. Thus, the initial position of the mouse cursor was the same on every trial within and across participants. The participant initiated the trial by clicking the left mouse button, after which one out of five possible cues appeared at the center of the screen. Simultaneously, the mouse cursor was unlocked allowing the participant to move the mouse cursor on the screen. After a 680-ms interval, the target was displayed at the left or right upper part of the screen. This provided ample time for the participant to initiate anticipatory mouse movements before the target occurred. Finally, the participant clicked on the target and the trial was terminated. A blank screen was displayed for 1,500 ms before the onset of the next trial (see Figure 1).

**Figure 2.**
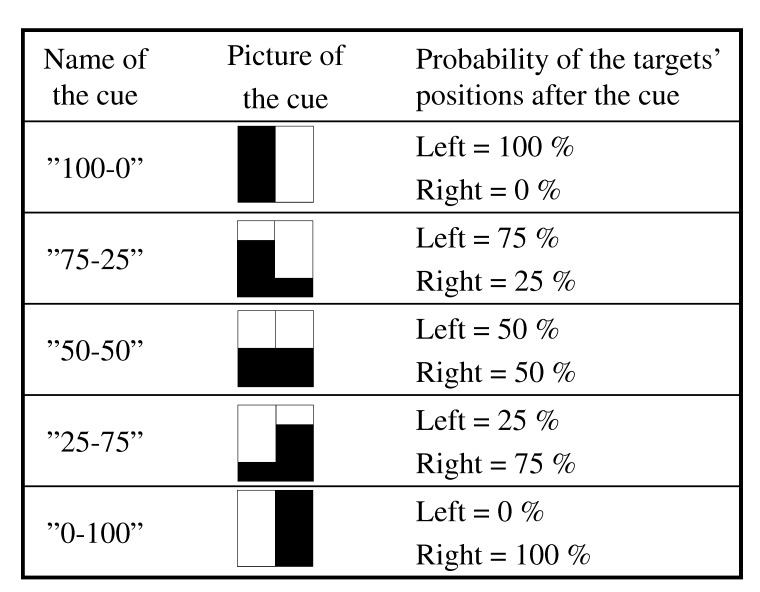
Overview of the five different cues and their relation to the probability of target position.

The five different cues and their corresponding probabilities that the subsequent target would occur on the left versus the right side of the screen are shown in Figure 2. Rather than using arbitrary symbolic cues, we chose cues whose physical appearance was designed to intuitively make sense as a visual signal for their specific predictive value regarding the upcoming target position. This was done because we wanted to make it moderately easy for the participants to develop a sense of the different cues’ predictive value, allowing for a potential effect of the degree of certainty to develop over the course of the experimental session.

### Instructions

At the beginning of the experimental session, instructions were given to the participants via an instruction screen on the computer monitor, which contained both text and figures. The participants were told to read the instructions thoroughly before starting. The instructions screen stated that the experiment investigated how quickly we respond to objects presented in different parts of the visual field and, further, that the participant should start each trial by clicking on the start box at the bottom of the screen and that, immediately hereafter, one of five possible figures (i.e., the cues) would occur. The five cues were displayed on the instruction screen, but with no reference to their predictive value. This was done in order to ensure that the participants paid attention to the cues during the experiment, while keeping them initially naďve of their different predictive values.^1^ Finally, the instructions stated that a green square would occur at the left or right upper part of the screen shortly after the black and white figure and that the participant’s task was to click as quickly as possible on the green square. No further instructions were given.

### Data collection and preprocessing

Throughout each trial, the following data were collected: (a) the time of the mouse-click on the start box, (b) the time at which the participant began moving the mouse, (c) the time of the mouse-click on the target square, and (d) the (*x, y*)-coordinates of the mouse position during the interval from the moment when the participant clicked on the start box (*start-click*) until s/he clicked on the target (*target-click*; sampling rate = 75 Hz). The sampled *x*- and *y*-coordinates were translated into a coordinate-system with its reference point (0, 0) in the mouse-cursor’s initial position (i.e., the center of the start box). The translated possible *x*-values span from -400 (the extreme left part of the screen) to 400 (the extreme right part of the screen), and translated possible y-values span from -35 (the extreme lower part of the screen) to 565 (the extreme upper part of the screen). In this coordinate system, *x* = 0 corresponded to the vertical midline of the screen, and thus (absolute) *x*-coordinate values reflected distance in pixels from the vertical midline. *Y*-coordinate values reflected distance in pixels from the horizontal line at the middle of the start-box.

### Experimental session

Each participant performed one experimental session of 290 trials, with a total duration of approximately 20 min. The 290 trials were divided into five successive blocks of 58 trials, which were enumerated as follows: the first 58 trials = Block 1, the next 58 trials = Block 2, etc. As illustrated in Table 1, the experimental structure was constructed to yield an equal number of trials with each of the five cues within each block and within the experiment as a whole. Therefore, there was a higher number of trials in the certain and the semi-certain condition compared to the uncertain condition. The order of the cues (and the associated target positions) was randomized without replacement within each block anew for each participant. Hence, trials from the three degree-of-certainty conditions also occurred in a random order within each block. However, the number of occurrences within each block of the different Cue × Target trial-types was constrained to assure that both the 75:25 ratio of valid versus invalid trials in the semi-certain condition and the 50:50 ratio of left versus right target side in the uncertain condition were present within each block (see Figure 2 and Table 1). Hence, the conditional probability of getting an invalid target outcome in the semi-certain condition (i.e., 25%) was the same within a given block as within the experiment as a whole. Likewise, the conditional probability of getting, say, a left target outcome in the uncertain condition (i.e., 50%) was the same within a given block as within the experiment as a whole. This design allowed us to look at the evolution of the effect of degree-of-certainty over the experiment by using Block as a factor (with five levels).

**Table 1.** Overview of the Block/Session Structure and the Independent Variables

Number of trials per block (total)	Cue	Target position	Degree- of-certainty
12 (60)	“100-0”	Left	Certain
10 (50)^a^	“0-100”	Right	Certain
9 (45)	“75-25”	Left	Semi-certain
9 (45)	“25-75”	Right	Semi-certain
3 (15)	“25-75”	Left	Semi-certain
3 (15)	“75-25”	Right	Semi-certain
6 (30)	“50-50”	Left	Uncertain
6 (30)	“50-50”	Right	Uncertain

^a^ The smaller number of trials with the “0-100” cue compared with the other four cues is due to a programming error. This was the case for all participants and did not affect the predictive value of the “0-100” cue (i.e., it was always followed by a right target).

### Dependent and independent variables

We wanted to investigate the emergence over time of differences in participants’ anticipatory behavior between the different degrees of certainty. Anticipatory behavior was operationalized by taking a “freeze-frame” immediately before the target occurred of the participant’s anticipatory hand movement on a given trial. This corresponded to measuring the *x*- and *y*-coordinates of the computer mouse 680 ms after cue onset. Hence, we had two dependent variables: the pre-target *x*-coordinate (the *x*-coordinate 680 ms after cue onset) and the pre-target *y*-coordinate (the *y*-coordinate 680 ms after cue onset). The pre-target *x*-coordinate reflected the spatial inclination of an anticipatory mouse movement toward one possible target location relative to the center of the screen. A high pre-target *x* value (whether positively or negatively signed) indicated proximity to one of the target locations (and, correspondingly, a high distance to the midline of the screen) whereas a pre-target *x* value of 0 indicated equidistance to the two possible target locations (and a positioning of the mouse cursor on the vertical midline of the screen). The pre-target *y*-coordinate indicated how close the mouse was to the target positions in the vertical plane immediately before the target occurred and thus it implicitly reflected how far the participants had moved the mouse overall on a given trial.

We used two independent variables for the analyses of this experiment: the block variable (described above) and the degree-of-certainty variable (described below). In this experiment, we were interested in if, and to what extent, participants made anticipatory movements toward the most likely target side (as indicated by the cue), but it was in principle irrelevant whether the cue indicated a high probability on the left or the right side. Thus, for the purpose of analyzing effects on anticipatory mouse movements (i.e., movements occurring before the target) we can consider the following two Cue × Target conditions functionally identical: (a) cue: “100-0”/target: left and (b) cue: “0-100”/target: right (certain condition). By the same token, when considering the time frame of the experimental trial before the target occurs, we can consider the following four Cue × Target conditions functionally identical: (a) cue:“75-25”/target: left, (b) cue: “75-25”/target: right, (c) cue: “25-75”/target: right, and (d) cue: “25-75”/target: left (semi-certain condition). Finally, we can consider the following two Cue × Target conditions functionally identical: (a) cue: “50-50”/target: left and (b) cue: “50-50”/target: right (uncertain condition). Hence, the eight levels of the Cue × Target conditions shown in Figure 2 were coded into the three-level degree-of-certainty variable as depicted in Table 1. Degree-of-certainty reflected how certain the participants could be about the position of the upcoming target *before* its occurrence, regardless of whether the target was cued to occur on the left or the right side and whether the target actually occurred on the left or the right side.

To be sure that it was unproblematic to collapse our anticipatory mouse movement data across upcoming target side, we performed a preliminary analysis comparing the magnitude of the anticipatory movements for left and right targets. We conducted ANOVAs of the effect of target side (left vs. right) on the absolute pre-target *x* values for each of the three degree-of-certainty conditions separately and found no effect of target side on pre-target *x*-coordinate in neither the certain condition, *F*(1, 16) = 1.50, *p* = .239; the semi-certain, *F*(1, 16) = 1.74, *p* = .196; nor the uncertain condition, *F*(1, 16) = 0.06, *p* = .809. It is worth noticing that this result is obtained when looking at absolute values of pre-target *x* (*x* = 0 corresponded to the vertical midline of the screen, so using the absolute pre-target values simply disregards the left-right direction and allows testing whether the magnitude of anticipatory movement in the horizontal dimension is different between left and right upcoming targets). We also conducted ANOVAs of the effect of target side (left vs. right) on the pre-target y values for each of the three degree-of-certainty conditions separately and found no effect of target side on pre-target *y*-coordinate in neither the certain, *F*(1, 16) = 0.25, *p* = .625; the semi-certain, *F*(1, 16) = 0.01, *p* = .927; nor the uncertain condition, *F*(1, 16) = 0.06, *p* = .809. Hence, there was no difference in the magnitude of anticipatory movements in neither the horizontal nor the vertical dimension between left and right targets.

After collapsing the conditions across target side we transformed the *x*-coordinates of the certain and the semi-certain conditions into a new scale in which positive values of *x* reflected a movement of the mouse in the direction of the most likely upcoming target position and negative values of *x* reflected a movement of the mouse in the direction of the alternative/unlikely position. This new scale was constructed by multiplying the *x*-values of the mouse movements occurring after the cues “100-0” and “75-25” with -1. In the uncertain condition, there was no more or less probable side so in this condition negative *x*-values simply reflected mouse movement toward the left side and positive *x*-values movement toward the right side.

## Results

### Movement initiation time

The movement initiation time was defined as the time from start-click until the participant began moving the mouse. The average movement initiation time was 193 ms (*SD* = 123), and the movement initiation time was shorter than 680 ms in 96.6% of the trials, signifying that participants started to move the mouse *before* the target was displayed on almost all the trials. There was no effect of degree-of-certainty on movement initiation time, *F*(2, 16) = 0.31, *p* = .739.

### Gradual emergence of probability-based modulation of anticipatory hand movements over the course of the experiment

**Figure 3.**
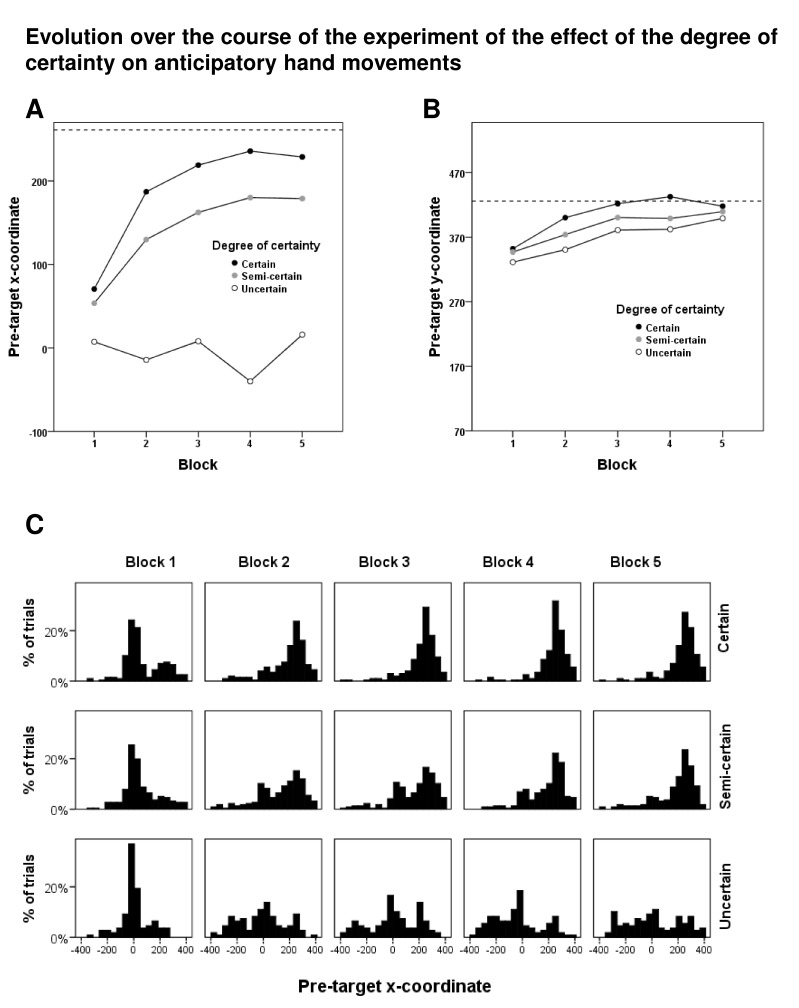
Evolution over the course of the experiment of the effect of the degree of certainty on anticipatory hand movements. In Sections A and B, the mean values of the pre-target *x*- and *y*-coordinates (i.e., the *x*- and *y*-coordinates 680 ms after cue onset) are shown for each of the three degrees of certainty as a function of block number. The dashed line in Section A shows the *x*-value of the left border of the target square, and the dashed line in Section B shows the *y*-value of the lower border of the target square. The histograms in Section C show the distributions of the pre-target *x*-coordinates for the combination of each of the five blocks with each of the three degrees of certainty.

Figure 3 shows the evolution of the effect of the degree of certainty on the pre-target *x*- and *y*-coordinates over the entire experimental session. The Degree-of-Certainty × Block interaction was significant for the pre-target *x*-coordinate, *F*(8, 64) = 6.38, *p* < .005, but not for the pre-target *y*-coordinate, *F*(8, 64) = 0.96, *p* = .479. There was a main effect of block on the pre-target *y*-coordinate, *F*(4, 32) = 4.09, *p* = .029.

### Effect of the degree of certainty on the pre-target hand position

**Figure 4.**
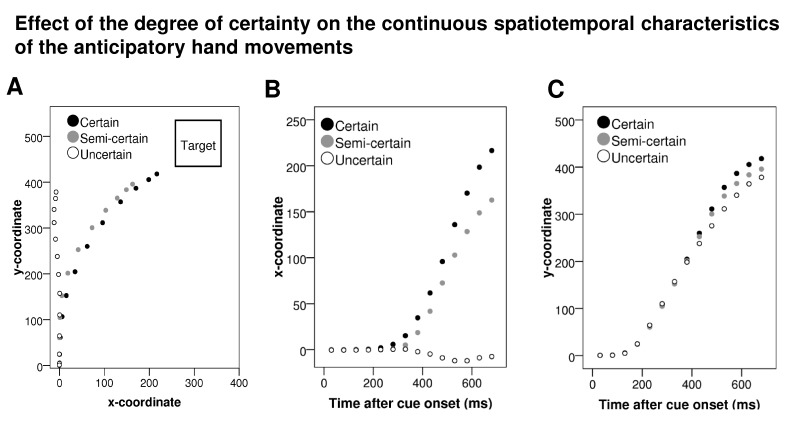
Effect of the degree of certainty on the continuous spatiotemporal characteristics of the anticipatory hand movements. The dots represent the mean values of *x*- and/or *y*-coordinates for the three different degrees of certainty at 14 consecutive time points, starting from 30 ms until 680 ms after cue onset with a 50-ms interval between the dots. The graph in Section A illustrates the effect of the degree of certainty on the mean continuous anticipatory hand movements and the target’s approximate position relative to these trajectories (data pooled from Blocks 2-5). In Sections B and C, respectively, the *x*- and *y*-coordinates are plotted separately as a function of time after cue onset.

As illustrated by the graphs in Figure 3, it is evident that the effect of the degree of certainty on the pre-target *x*- and *y*-coordinate is particularly present from the second block on. Therefore, we used the pooled data from Blocks 2-5 for the construction of a graphical representation of the overall effect of degree-of-certainty on the mean anticipatory hand movements and for statistical inference on this effect. Figure 4 (Sections A-C) shows the mean continuous anticipatory mouse trajectory for the three degrees of certainty pooled across Blocks 2-5. The *x*- and *y*-values used for the inferential statistics reported below correspond to the last three data-points at the outer right part of the graphs in Figure 4 (Sections B and C, respectively) and to the three uppermost data-points in Section A of Figure 4. In the data pooled across Blocks 2-5, there was a main effect of degree-of-certainty on the pre-target *x*-coordinate, *F*(2, 16) = 45.80, *p* < .005, with the highest pre-target *x*-value in the certain condition (*M* = 218, *SD* = 34), medium in the semi-certain condition (*M* = 163, *SD* = 70), and lowest in the uncertain condition (*M* = -8, *SD* = 41). Planned comparisons revealed that the difference between the certain and semi-certain conditions was significant, *t*(8) = 2.90, *p* = .020, and that the difference between the semi-certain and uncertain conditions was significant, *t*(8) = 5.50, *p* = .001. There was also a main effect of degree-of-certainty on the pre-target *y*-coordinate, *F*(2, 16) = 14.12, *p* < .005, with the highest pre-target *y*-value in the certain condition (*M* = 418, *SD* = 30), medium in the semi-certain condition (*M* = 396, *SD* = 40), and lowest in the uncertain condition (*M* = 378, *SD* = 40). Planned comparisons revealed that the difference between the certain and semi-certain conditions was significant, *t*(8) = 3.94, *p* = .004, and the difference between the semi-certain and uncertain conditions was significant, *t*(8) = 2.47, *p* = .039.

### Continuous spatiotemporal characteristics of the anticipatory hand movements

The graphs in Figure 4 suggest the existence of two phases in the participants’ anticipatory hand movements occurring from start-click to target occurrence. First, there is an initial upward going movement, which was not modulated by the degree of certainty, starting approximately 200 ms after the cue (see Section C of Figure 4): The *y*-coordinate 180 ms after cue onset (the fourth time point from the left in Figure 4, Section C) was non-different from zero in all three degree-of-certainty conditions - certain condition: *t*(8) = 2.07, *p* = .073;semi-certain condition: *t*(8) = 2.11, *p* = .068; uncertain condition: *t*(8) = 2.21, *p* = .058 - but at 230 ms after cue onset (the fifth time point from the left in Section C of Figure 4), the *y*-coordinate was significantly different from zero in all three degree-of-certainty conditions - certain condition: *M* = 61, *SD* = 79, *t*(8) = 2.32, *p* = .049; semi-certain condition: *M* = 61, *SD* = 74, *t*(8) = 2.46, *p* = .040; uncertain condition: *M* = 65, *SD* = 79, *t*(8) = 2.45, *p* = .040. Time point 230 ms was the first of five immediately succeeding time points from Figure 4 (Section C) were the *y*-coordinate was significantly different from zero in all three degree-of-certainty conditions (*t*-tests not shown for time points 280 ms, 330 ms, 380 ms, and 430 ms). Second, at around 400 ms after cue onset, a second phase can be observed where the effect of the degrees of certainty on anticipatory hand movements starts to appear. This is reflected both in movement along the *x*- and the *y*-axis: (a) for the *x*-coordinate, the effect of degree-of-certainty was non-significant 380 ms after cue onset (the eighth time point from the left in Section B of Figure 4), *F*(1.11, 8.90) = 3.63, *p* = .087, but at 430 ms after cue onset (the ninth time point from the left in Section B of Figure 4), it was significant, *F*(1.01, 8.78) = 6.23, *p* = .033; and (b) for the *y*-coordinate, there was no effect of degree-of-certainty 380 ms after cue onset (the eighth time point from the left in Section C of Figure 4), *F*(2, 16) = 0.70, *p* = .512, but at 430 ms after cue onset (the ninth time point from the left in Figure 4, Section C), the effect was significant, *F*(2, 16) = 4.90, *p* = .022. For both the *x*- and the *y*-coordinate, time point 430 ms was the first of five immediately succeeding time points from Figure 4 (Sections B and C, respectively) where the effect of degree-of-certainty on the coordinate was significant (*t*-tests not shown for time points 480 ms, 530 ms, 580 ms, and 630 ms).

### Individual differences in anticipatory hand movements in the uncertain condition

**Figure 5.**
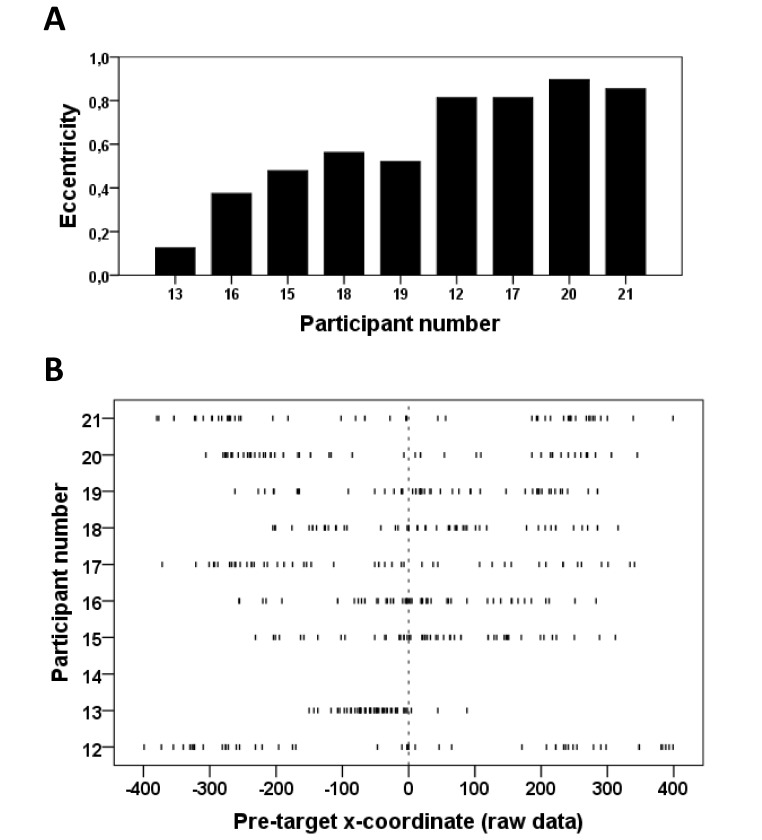
Section A. Plot of the eccentricity values in the uncertain condition for single participants. Section B. Scatter plot of pre-target *x*-coordinates for all trials in the uncertain condition for each participant. Each vertical line corresponds to a single trial. Note that the results of Participant number 14 do not appear because he was excluded (see Methods section).

Because of the broad distribution of the pre-target *x*-coordinates in the uncertain condition (Figure 3, Section C, bottom row), we decided to look at single participants’ contributions to the different ranges of values in this condition. To do this, we created an *eccentricity* variable using data from Blocks 2-5. We assigned a value of 0 for trials in which the pre-target *x*-coordinate ranged from -100 to 100 and a “1” for trials in which the pre-target *x*-coordinate was lower than -100 or higher than 100. Hereafter, we treated eccentricity as a continuous variable and calculated the mean eccentricity for each participant. This gave a number indicating on how large a proportion of the trials a given participant’s hand position was in the proximity (on the horizontal axis) of the one of the possible target locations immediately before the target occurred and, inversely, how often the participant’s hand position stayed around the *x* = 0 line. An eccentricity value close to 1 indicated that the participant had made anticipatory hand movements directed toward one of the possible target locations on the majority of the trials, and an eccentricity value closer to zero indicated that the participant had kept the mouse around the center of the screen (i.e., around *x* = 0) on a larger proportion of the trials. Section A of Figure 5 shows the eccentricity values for each of the nine participants. Because the eccentricity value does not distinguish between movements to the left and right side, Figure 5 (Section B) shows a scatter plot where the pre-target *x*-coordinate of single trials in the uncertain condition is plotted for each participant. Examination of this graph allows determining if a high eccentricity value for a given participant is due to anticipatory hand movements that are consistently biased towards one side or to random movements towards both sides. The latter appears to be the case for most participants, although there is a visible leftward bias for Participant number 13.

## Discussion

First of all, our mouse tracking data revealed that participants spontaneously engaged in anticipatory hand movements in all three degree-of-certainty conditions. These spontaneous anticipatory hand movements are evidenced by the anticipatory trajectories (cf. Figure 4) in conjunction with the very low movement initiation times. An interesting finding is that this tendency to spontaneously engage in anticipatory behavior was equally present for the three degrees of certainty. It is evident from the *y*-coordinate data shown in Section C of Figure 4 that participants moved the mouse upward even in the uncertain condition. Hence, even when participants did not know which side to go to, rather than just awaiting passively to react to the target when it occurred, participants started moving the mouse in anticipation of the target’s arrival. This finding is in line with results from another recent study that used computer mouse tracking to investigate anticipatory behavior (Dale et al., 2012). Dale et al. implemented a modified serial reaction time task where participants moved the computer mouse around on the screen to click on sequentially occurring stimuli appearing in one of four positions located at the four corners of the screen. In different conditions, the sequential ordering of positions was more or less structured, making the position of the next stimulus more or less predictable. Dale et al. observed that, when the position of the next stimulus was unpredictable, rather than passively awaiting the occurrence of the next stimuli, participants often adopted a “centering strategy” consisting in “an optimal anticipatory positioning close to [all] the possible future stimuli” (p. 204). The present findings and those of Dale et al. indicate that participants have a strong tendency to spontaneously engage in anticipation of upcoming events even under conditions of uncertainty regarding the future outcome.

The participants’ tendency to engage in anticipatory movements in our uncertain condition could reflect a carryover of strategy from the other two conditions, and it is possible that this result would have been different if we had manipulated the degree-of-certainty variable across blocks^2^ instead of mixing the degree-of-certainty conditions in a random order within each block . However, Dale et al. (2012) also observed anticipatory “centering” movements under conditions of uncertainty although the overall predictability of a sequence of stimuli was manipulated block-wise in their experiment. Note moreover that classical spatial cuing experiments also typically manipulate cue validity on a trial-to-trial basis, so these are also subject to potential carryover effects.

Whereas the tendency to initially engage in anticipatory hand movements did not differ between the different conditions in our experiment, the probability/degree of certainty concerning the upcoming target position significantly affected the spatial characteristics of the anticipatory hand movements. The plots of the evolution over the course of the experiment of the effect of degree-of-certainty on the pre-target x- and *y*-coordinates (Figure 3, Sections A and B) reveal that there was a rapid emergence of probability-based modulation of participants’ hand position immediately before the target. Hence, presenting participants with a constant probabilistic relationship between given cues and given upcoming target positions resulted in the recurring probability structure of the “experimental world” being rapidly (i.e., within the first 58 trials) assimilated by the participants in a way that caused them to modulate their anticipatory hand movements correspondingly. This rapid emergence of explicit behavioral effects of the exposure to an implicit statistical structure is in line with findings from studies on statistical learning (e.g., Dale et al., 2012; Kirkhamet al., 2002, 2007). Although the effect of the degree of certainty on participants’ pre-target hand position appeared already after the first block and stayed rather stable from the second block, there seemed to be a small change over the course of the experiment in the shape of the underlying distribution of trials. Based upon visual inspection of the distributions of the pre-target *x*-coordinates in the semi-certain condition (see Figure 3, Section C, second row), there appears to be a gradual evolution from a somewhat bimodal shape of the distribution in Block 2 toward a more unimodal distribution in Block 5. This pattern might suggest that the consolidation over time of probability-based anticipation have complex effects on behavior that are not detectable in mean values. However, due to the relatively small number of observations in the Block × Degree-of-certainty subsets of data we cannot conclude statistically on this apparent change in the shape of the distribution.

As depicted in Figure 4 (Section A), we found that when the cue predicted the upcoming target position with 100% certainty, then participants made anticipatory hand movements relatively directly toward the predicted position. This is in contrast to the (averaged) anticipatory movement trajectories observed in the uncertain condition, which stayed in the center with equal distance toward the two targets. When we compare the averaged anticipatory trajectories of the certain and semi-certain conditions, there is a lateral shift toward the alternative/unlikely target side in the semi-certain relative to the certain condition, reflected in the pre-target *x*-coordinate being lower in the semi-certain than in the certain condition. This finding of a behavioral difference between the certain and the semi-certain condition is consistent with findings from spatial cuing studies showing that semi-certainty (70 to 90%) yields higher reaction times than complete certainty of outcome (Drazin, 1961; Eriksen & Yeh, 1985; Geng & Behrmann, 2005). In the context of the present study, this lateral shift can be taken as an indication of a higher spatial attraction toward the alternative/unlikely target side in the semi-certain than the certain condition. This finding is in line with a more general picture that has emerged from previous mouse tracking studies: Higher degrees of ambiguity/uncertainty in a dichotomous decision task (McKinstry et al., 2008) and a phonological processing task (Spivey et al., 2005) have been associated with higher spatial inclination of hand movements toward an alternative choice category.

A potential methodological limitation of this study concerns the fact that the cues were not counterbalanced, that is, the darker side of the cue always coincided with the high-probability side in the certain and semi-certain conditions. We cannot exclude that the differences observed in anticipatory hand movements between our conditions were the result of a tendency to automatically move the mouse towards the darker side. However, effects of a degree-of-certainty manipulation on anticipatory hand movements similar to the ones reported here were observed in another study in which cues with a darker and a lighter side were counterbalanced across participants such that the lighter side of the cue corresponded to the high-probability side for half of the participants (Bruhn, Huette, & Spivey, 2012). It is therefore unlikely that the effects observed in the current study were the result of a bias of inherently induced movement towards the darker side.

It has been proposed that the continuous motor output sampled with computer mouse tracking can be conceived of as a two-dimensional projection of the ongoing perceptual and cognitive dynamics involved in accomplishing the current task (Spivey, 2007; see also Freeman, Dale, & Farmer, 2011, and Magnuson, 2005). Hence, the anticipatory hand movements measured in this study can be seen as a reflection of the underlying (psychological and, possibly, neural) anticipatory processes. From this perspective, the higher spatial attraction of the anticipatory hand movements toward the alternative target side in the semi-certain condition could be taken to suggest that when we can only be semi-certain about the outcome of two alternative upcoming events we do not “go” entirely for the most likely event, but rather engage some resources in anticipating the unlikely event also (see Bruhn et al., 2012, for further support and discussion of this interpretation).

A central issue addressed by previous mouse tracking research has been whether a higher average spatial attraction toward an alternative location is indeed the result of a continuous, graded modulation of spatial attraction on a trial to trial basis, rather than being the result of the presence of a subset of trials with very high spatial attraction toward the alternative side in the high-attraction condition (see e.g., Spivey et al., 2005). In the context of the present study, this latter scenario would be associated with a bimodal distribution of the pre-target *x*-coordinates in the semi-certain condition. As noted above, such bimodality may be present in (a subset of) the semi-certain trials, but cannot be statistically tested for because of too few observations.

## Continuous spatiotemporal characteristics of anticipatory behavior

In addition to the “freeze-frame” of anticipatory hand movements, conveyed through the pre-target *x*- and *y*-coordinates, we also presented results of the continuous spatiotemporal dynamics of anticipatory hand movements over the course of the entire anticipatory period (Figure 4, Sections A-C). These data provide a continuous depiction of anticipatory behavior at the crucial time frame of when it occurs and contains richer information on anticipatory processes than what can be obtained from reaction time studies. The analyses carried out on the continuous trajectories in Figure 4 suggested that there were two phases in the participants’ anticipatory hand movements occurring from start-click to target occurrence. The very early vertical movement observed in the first phase, which is not modulated by the degree of certainty, is probably a reflection of anticipatory processes occurring already before the cue is displayed. Indeed, before the participants initiated a trial they already knew that the target would occur in the upper part of the screen and, hence, the preparation of a simple upward going momentum would be functional independently of the nature of the subsequence cue. This type of behavior is in fact an excellent example of how participants spontaneously use available information to anticipate upcoming events independently of whether this information is what the experimenter manipulated and intended for the participants to base their anticipation on. The second phase, starting around 400 ms after cue onset, marked the time point at which a modulation of participants’ anticipatory behavior corresponding to the probabilistic information of the cue started to emerge. What should be noted about these findings is not so much the absolute time-course of the transition from the first to the second phase of anticipatory behavior, as this is possibly specific to the task constraints of the current experiment. Indeed, Gibson and Kingstone (2006) found an effect of four different types of spatial cues on reaction time after only 250 ms, that is, some hundred milliseconds before the effects of the cue was visible in the continuous anticipatory hand movements in our experiment. Rather, what is interesting about the current results is that the anticipatory hand movements of the first phase (from approx. 200 to 400 ms) indicate the existence of anticipatory processes that are not dependent on the information conveyed by the cue and that are probably initiated already before the cue was displayed.

## Individual differences in anticipatory behavior in the uncertain condition

When we looked beyond the mean value of the pre-target *x*-coordinate in the uncertain condition, the underlying distribution of trials revealed that the mean value of x 0 is actually the result of a mixture of trials where participants’ pre-target hand position was around the *x* = 0 line and of trials where it was closer to one of the two possible target sides (Section C of Figure 3, third row, Blocks 2-5). The individual eccentricity values shown in Figure 5 (Section A) revealed that participants did not contribute evenly to the “bumps” of pre-target x-values around 0, on the one hand, and of extreme (positive and negative) pre-target *x*-values, on the other hand. Some participants (plotted in the right part of the graph in Section A of Figure 5) have eccentricity-values close to 1, indicating that they made anticipatory hand movements toward one of the target position on most trials in the uncertain condition. Hence, these participants exhibited a “chance-taking” strategy in the uncertain condition, where they moved the mouse randomly toward one of the target positions. Other participants (those with eccentricity values closer to 0.5, plotted in the left part of the graph in Figure 5, Section A) exhibited a more mixed strategy, “taking a chance” on some trials and being more “cautious” in their anticipatory hand movements on other trials (i.e., staying around the *x* = 0 line and awaiting the actual outcome of target occurrence). Thus, there were notable individual differences in the way participants anticipated when faced with uncertainty of future outcome. Dale et al. (2012) also found individual differences in anticipatory behavior under conditions of uncertainty. Future studies could further investigate this intriguing finding of individual differences.

## Implications for probabilistic spatial cuing

Studies on probabilistic spatial cuing have traditionally used measures of reaction time and accuracy to infer on processing that occurs before these measures are collected (e.g., Downing, 1988; Eriksen & Yeh, 1985; Geng & Behrmann, 2005; Gottlob, Cheal, & Lyon, 1999; Posner, 1980, among many others). The mouse tracking approach used in the present study provided novel insight into anticipatory processes that has implications for probabilistic spatial cuing. Specifically, the findings presented here suggest that: (a) anticipatory processes directed at the upcoming target can be initiated *before* a cue is presented, (b) the condition typically presumed to be “neutral” in spatial cuing paradigms (i.e., the condition where no information on the location of the upcoming target is given, here referred to as the *uncertain condition*) can induce unique anticipatory processing of its own, and (c) as also supported by reaction time studies, a “valid cue” cannot necessarily be considered a unitary concept that can be unproblematically compared across different (high) degrees of certainty because conditions of 100% certainty (i.e., using a 100%-valid cue) and semi-certainty (using a generally valid cue) appears to engender differential anticipatory processes.

## Anticipation and computer mouse tracking

The interest for understanding anticipatory/predictive processes in cognition has considerably increased over the recent years (see e.g., Bubic et al., 2010; Pezzulo et al., 2007). From this perspective, the instantiation of a simple behavioral paradigm that allows tapping into anticipatory processes as they occur represents a valuable methodological contribution. The anticipatory hand movement data presented by this and other recent studies (Bruhn et al., 2012; Dale et al., 2012) suggest that the application of computer mouse tracking to the study of anticipation is a promising approach that could be favorably applied by future studies to investigate anticipatory processes in different contexts. Computer mouse tracking represents a supplementary/alternative method to other methods that allow tracking processes occurring before an expected target such as eye-tracking and brain imaging techniques with high temporal resolution (e.g., electroencephalography/ magnetoencephalography; EEG/MEG). As a behavioral measure, hand movements cannot substitute direct measures of brain activity. However, the mouse tracking methodology is a much cheaper and more accessible single tool to study anticipatory processes in real-time than both eye-tracking and EEG/MEG. Moreover, in comparison to eye-movements, which are ballistic and therefore do not provide a genuinely continuous real-time measure, mouse movements provide continuous time-course data on a single trial level (see Freeman et al., 2011; Magnuson, 2005; Spivey, 2007, for a discussion of this). On the other hand, eye-movements have a lower threshold for execution than hand movements, and might therefore be able to capture subtler and more transiently occurring processes than hand movements. Hence, future research would benefit from combining anticipatory hand movements with eye-movements and measures of anticipatory brain activity for a more complete understanding of anticipatory processes.

## Footnotes

1 In a pilot study, in which the cues were not displayed as a part of the instruction screen, the majority of the participants reported to have ignored the cues, thinking that they were simply “distractors”.

2 That is, having one block with only certain trials, one block only all semi-certainty trials, and one block with only uncertain trials, with the order of certain, semi-certain, and uncertain blocks counterbalanced across participants.

## Author Note

I am grateful to Bo Sommerlund for his help with programming of this experiment and development of software for data analysis, to Klaus Bćrentsen and Risto Näätänen for comments on an earlier version of this manuscript, and to Maria Nordfang for comments on the current version of our article.
